# Understanding patient activation and adherence to nebuliser treatment in adults with cystic fibrosis: responses to the UK version of PAM-13 and a think aloud study

**DOI:** 10.1186/s12913-019-4260-5

**Published:** 2019-06-24

**Authors:** Jie Gao, Madelynne Arden, Zhe Hui Hoo, Martin Wildman

**Affiliations:** 1 Centre for Behavioural Science and Applied Psychology, Sheffield Hallam University, Sheffield, UK; 2 School of Health and Related Research (ScHARR), University of Sheffield, Sheffield, UK; 3 Sheffield Adult Cystic Fibrosis Centre, Northern General Hospital, Sheffield, UK

**Keywords:** Patient activation, PAM-13, Cystic fibrosis, Objective medication adherence, Think-aloud

## Abstract

**Background:**

Patient activation refers to patients’ knowledge, skills, and confidence in self-managing health conditions. In large cross-sectional studies, individuals with higher patient activation are observed to have better health outcomes with the assumption that they are more engaged in health self-management. However, the association between patient activation and objectively measured self-care indicators in individuals can be inconsistent. This research investigated the role of patient activation as measured by the UK Patient Activation Measure (PAM-13) in adults with Cystic Fibrosis (CF). The aims were twofold: to explore how adults with CF interpret and respond to the PAM-13; and to investigate the association between PAM-13 and objectively measured nebuliser adherence in UK adults with CF.

**Methods:**

This article describes two studies which examined the PAM-13 from different perspectives. Study 1 comprised ‘think aloud’ interviews with 15 adults with CF. The data were analysed using an a priori coding framework. Study 2 examined the association between PAM-13 and objectively measured nebuliser adherence in 57 adults with CF.

**Results:**

Study 1 showed that adults with CF encountered several difficulties while completing the PAM-13. The difficulties were related to understanding how to interpret aspects of CF in order to respond *(*i.e., control over the condition, ability to exercise) and item wording. Some adults with CF responded to the PAM-13 in an optimistic way in relation to what they thought they should do rather than what they actually do. These findings were echoed by the results of Study 2, which showed that PAM-13 scores were not significantly correlated with objective medication adherence in a different sample. This article synthesises the results of both studies, providing insights into influences and associations of patient activation as measured by the UK PAM-13 in adults with CF.

**Conclusions:**

There were some significant difficulties created by the wording of the UK PAM-13 for adults with CF. This may partly explain the finding that PAM-13 scores were not related to objectively measured nebuliser adherence in this study. The UK PAM-13 would benefit from further research to verify its validity and reliability in different patient populations against objective measures of behaviour rather than simply self-report.

## Background

Patient activation is a concept that is used to describe a patient’s knowledge, skills, and confidence in managing his or her health conditions [1]. Conceptually, patient activation describes the characteristics of patients who are more likely to participate as active members of their care team [2], and engage in the behaviours that maintain their health such as adherence to medication [1].

The Patient Activation Measure (PAM) was developed using the findings from an expert consensus panel and patient focus groups to identify and define the questionnaire domains and subsequent testing and refinement using psychometric analysis [1]. Patient activation draws on concepts such as health locus of control [3], self-efficacy in managing health behaviours [4] and readiness to change health behaviours [5], but is not condition or behaviour specific. The original Patient Activation Measure (PAM) has 22 items and a 13-item version has also been developed (PAM-13) [6]. Both utilise a four-point Likert scale of agreement-disagreement to respond to each item. PAM is scored on a scale from 0 to 100 from which four levels of activation have been identified: Level 1 (0.0–47.0) low activation suggesting that the person does not yet understand their role in healthcare; Level 2 (47.1–55.1) indicating that the person does not yet have the knowledge and confidence to take action; Level 3 (55.2–72.4) indicating that the person is beginning to engage in positive health behaviours; Level 4 (72.5–100) indicating that the person is proactive and engaged in recommended health behaviours [7].

There is some self-report evidence that individuals with higher patient activation are more engaged in self-managing their long-term conditions, thereby having better health outcomes and care experiences. For example, Mosen et al. [8] described the results from a cross-sectional survey of over 4000 people with a range of chronic health conditions. They reported positive relationships between PAM-22 scores and self-reported measures, including self-management behaviours, use of self-management services, and medication adherence as well as patient satisfaction, quality of life, and physical and mental functional status. Barker et al. [9] analysed a database of English NHS and found that high patient activation was associated with lower healthcare utilisation and less wasteful use across primary and secondary care. Kinney et al. [10] conducted a systematic review of published literature on the association between the PAM and hospitalization, emergency room use, and medication adherence among chronically ill patient populations. The review indicated that lower PAM scores were associated with higher rates of hospitalisation and use of emergency room services but that the relationship with adherence to medication was inconclusive.

The PAM has been widely used in the US as a tool to measure and target support for patient engagement and self-management, particularly with regard to long-term conditions. It has also been translated and validated for use in several languages and countries [11].

The PAM was introduced into UK in 2005. Ellins and Coulter [12] validated the 22-item PAM in a National Telephone Survey study in the UK. They anglicised some key terms and phrases to better suit the UK population. Subsequently, the validated PAM (mostly 13-item version) has been used as an outcome measure to evaluate intervention programmes in the UK [13–16]. Use of the PAM is becoming much more frequent in the UK, and NHS England has agreed a five-year licence to use the PAM-13 with up to 1.8 million people across the NHS from 2016 as part of its ‘Self-Care programme’ [17] which seeks to support people living with long-term health conditions to better manage their own health. Five Clinical Commissioning Groups (CCGS) and one disease registry are currently using PAM across a range of projects [17]. However, some concerns have been raised about the appropriateness of the UK-version of PAM in these contexts [17].

Kidd et al. [18] examined the content and wording of the PAM-13 and its ease of use in UK stroke survivors. Some patients reported that they found it difficult to respond to certain items. For example, the item “*I understand my health problems and what causes them*” actually indicates at least two distinct health issues (*A*. understanding the health problem and *B*. understanding what causes the health problem), which the patients reported that they might have different responses. Kidd et al. [18] also found that patients’ PAM-13 scores did not necessarily match the narratives which patients provided about their activation levels.

Armstrong et al. [19] conducted an independent evaluation of the feasibility of using the PAM-13 in the NHS in England and identified some potential problems. For example, some health coaches reported that their patients struggled with the meaning of some items of the PAM-13 and found some items irrelevant to their health conditions. The health coaches suggested that patients may not engage with the PAM-13 properly. Moreover, Armstrong et al. [19] found that the PAM-13 seemed to be problematic for patients with multiple co-morbidities, as their responses tended to vary depending on which health condition they were thinking about when completing the measure. The generalised wording of the PAM-13 makes it difficult for patients with multiple co-morbidities to respond with certainty as they may want to respond differently depending on which one of the co-morbid conditions they choose to focus on. Nonetheless, Blakemore et al. [20] used the PAM-13 in older people with multiple co-morbidities in the UK and found that the PAM scores were associated with the number of self-reported co-morbidities and the perceived impact of those comorbidities. The use of PAM-13 in UK patients with multiple co-morbidities is therefore unclear.

Cystic fibrosis (CF) is a life-limiting genetic condition with multiple effects on the body. Acute and chronic lung infections are common in adults with CF and adherence to medication is crucial to reduce exacerbations and preserve lung function. Nebuliser treatments are prescribed as preventative treatment, however as with other long-term conditions adherence is low [21] with objective data indicating that median adherence is only 36% [22]. Many people with CF also have co-morbidities including diabetes [23]. Patient activation and resultant engagement with self-management is therefore a potentially important concept in CF care.

In order to investigate the role of patient activation as measured by the UK PAM-13 in adults with CF, two studies with different foci were conducted separately in the UK. The first study aimed to explore how adults with CF interpret and respond to the UK version PAM-13. The second study analysed data from a pilot randomised controlled trial to examine the association between the PAM-13 and objective measures of nebuliser adherence in adults with CF at baseline and five-month follow-up. This article synthesises the findings of both studies in order to provide multifaceted perspectives on the results obtained when patient activation is measured by the UK PAM-13 in adults with CF.

## Methods

### Design

This article combines two studies of patient activation which were carried out separately in the UK. Each study demonstrates a different aspect of the application of the UK PAM-13 in adults with CF.

Study 1 utilised the think-aloud technique to investigate how adults with CF understand and respond to the PAM-13. Think-aloud is a cognitive interview technique that enables participants to verbalise thoughts that would normally be silent [24]. It is an established technique to investigate an individual’s response process when completing a questionnaire [25].

Study 2 investigated the association between the PAM-13 and objectively measured adherence to nebuliser medication in adults with CF measured at two time points as part of a pilot randomised controlled trial of an intervention (CFHealthHub) [26].

### Participants

For study 1, participants were recruited from a Cystic Fibrosis centre in the North of England. The Inclusion criteria were that they were English-speaking patients with CF, aged 16 years and above who were using an Etrack® nebuliser (Pari) which collects objective data about the date and time that treatments are taken. Participants were excluded if they were pregnant, post-transplant, or on the active transplant list or in the palliative phase of disease.

**Fig. 1:**
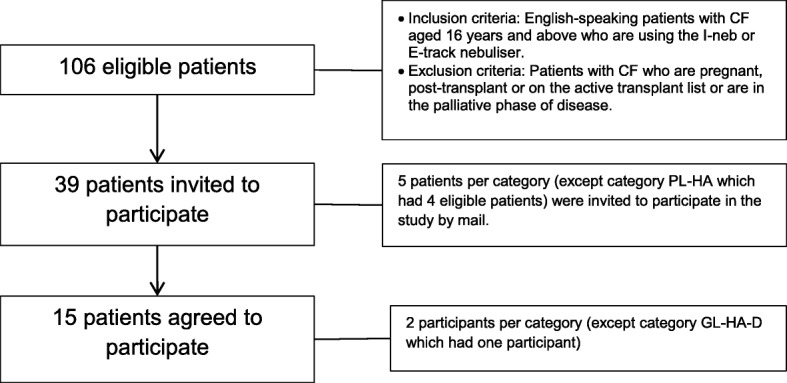
Procedure of participant recruitment of study 1

One hundred six patients met the above criteria. We contacted 39 participants who represented a range of participant characteristics based on their objective adherence level (high/low^1^), lung function (good/poor^2^) and with/without a co-morbidity of diabetes. Fifteen patients (6 females and 9 males) consented to participate (see Fig. 1 and Table 1).

**Table 1:** Participant characteristics of each category for study 1

Category code	Patient characteristics	NO. of participants
GL-HA	Good lung function, High adherence, Without diabetes	2
GL-HA-D	Good lung function, High adherence, With diabetes	1
GL-LA	Good lung function, Low adherence, Without diabetes	2
GL-LA-D	Good lung function, Low adherence, With diabetes	2
PL-HA	Poor lung function, High adherence, Without diabetes	2
PL-HA-D	Poor lung function, High adherence, With diabetes	2
PL-LA	Poor lung function, Low adherence, Without diabetes	2
PL-LA-D	Poor lung function, Low adherence, With diabetes	2
Total		15

For study 2, participants were recruited from two other Cystic Fibrosis centres in the UK (one in the midlands and one in the south of England) to take part in a pilot study of an intervention to increase adherence to nebulised medication [26]. Eligible participants were aged 16 years and over, taking or willing to take inhaled medication via a nebuliser with data recording and transfer capability. We excluded those who were: post-lung transplant; on the active transplant list; receiving palliative care; lacking capacity for informed consent; or, using other devices to take their inhaled treatment which did not provide objective adherence data. Four hundred thirty patients were screened and 135 (31.4%) were eligible. Ninety-five were able to be contacted and 64 (Mean age = 29.7, 56% male) consented and provided data at baseline (see Table 2 for full baseline characteristics). Two participants died, one withdrew consent, two were lost to follow-up, and two withdrew from adherence data collection over the course of the study so that 57 participants provided data at follow-up.

**Table 2:** Baseline characteristics of study 2 participants

	Baseline
Age	
Mean (SD)	29.7(11.5)
Median (IQR)	27(21, 36)
Sex	
Male	36 (56%)
Female	28 (44%)
FEV 1% Predicted	
Mean (SD)	57.3 (21.3)
Median (IQR)	49.6 (41.9, 76.7)

### Measures

#### The Patient Activation Measure (UK PAM-13)

Patient activation was measured using the PAM-13® (Insignia, UK version). This consists of 13 items, with a 4-point Likert scale for each item from agree to disagree. Scoring of the questionnaire was completed using the scoring algorithm provided by Insignia.

#### Medication adherence

Objective adherence was measured using an Etrack® nebuliser (Pari). The data were used to calculate the percentage of treatments taken relative to the number prescribed. For Study 1, the most recent 12-month of data in the period from 01 Jan 2016 to 31 Mar 2017 were used. For Study 2, the data over 2 two-week^3^ periods at baseline and five-month follow-up, respectively, were used.

### Procedure

In Study 1, eligible participants were invited to interview. Interviews took place either at the hospital or at the patient’s home. Participants were provided with information about the study and provided written consent to take part. All interviews were audio-recorded and transcribed and any identifiable information was removed. Names were replaced by a code which reflected level of adherence, lung function and whether or not they had diabetes (see Table 1). Each interview took approximately half an hour.

During the think aloud procedure, participants were asked to speak everything that they were thinking about while completing the questionnaire with minimum interference from the researcher. Participants were not required to reflect on their thoughts during thinking aloud. Rather they were asked to report their thoughts concurrently. As a result, the authentic thoughts of the participants were recorded for analysis.

Instructions on how to ‘think-aloud’ were provided to the participant. They then watched a video clip of a think-aloud interview in response to a different questionnaire. Next, a warm-up questionnaire was given to the participant to practice ‘thinking aloud’. During the process, the researcher checked whether the participant understood and could perform ‘thinking aloud’ properly and answered any questions as necessary. The participant then completed the PAM-13 while ‘thinking-aloud’. The researcher did not interrupt unless the participant paused for more than 10 sec when the researcher asked the participant to “keep talking please”. After the participant completed the PAM-13, the completed questionnaire was collected and a few follow-up questions were asked, such as “what do you think about the questionnaire? Which statements do you find most difficult to respond to?”. At the end of the interview, participants were debriefed and provided with contact information of the researchers.

In Study 2, measurements were taken as part of the procedures in a pilot randomised controlled trial of an intervention (CFHealthHub) to increase nebuliser adherence in adults with CF, which is described elsewhere [26].

Participants who met the inclusion criteria consented to take part and completed a battery of questionnaires including the PAM-13 at baseline. They were then provided with an E-track nebuliser and Qualcomm hub which they plugged in at their home and this sent data about the date and time of nebulised treatments to the CFHealthHub web platform. They then completed the questionnaire battery including the PAM-13 at five-month follow up.

Mean objective adherence data (number of treatments taken/number of treatments prescribed) was calculated at baseline (14 days post-consent with the PAM measured at consent) and follow-up (14 days preceding the completion of follow-up questionnaires).

### Analysis

In Study 1, the audio-recording of the interviews were transcribed verbatim. The transcripts were analysed using an a priori coding framework which classifies the data into codes based on suitable question, problematic content and misinterpretation (see Table 3 for details). This coding framework has been used in previous cognitive interview studies showing it is a sound framework for evaluating questionnaires [31, 32]. The data was firstly coded in accordance with the framework. Subsequently, the data under each code were analysed to generate themes representing the rationale for the initial coding [31]. The codes and the corresponding themes were discussed between two researchers (JG and MA) and a consensus was reached.

**Table 3:** The a priori coding framework

Coding framework	
Code 1 Suitable item	No problems emerged
Code 2 Problematic content	Participants questioned about the item content or wording or scale categories.
Code 3 Misinterpretation	Participants misunderstood the items or their verbal responses did not justify their choices.

In study 1 and 2, PAM-13 scores and levels (1–4) were calculated for participants at baseline and follow-up using the scoring algorithm provided by Insignia. Percentage objective nebuliser adherence was calculated at baseline and follow-up and then categorised as being very low (< 25%), low (25.1–50%), moderate (50.1–75%) or high (> 75%) according to Hoo et al. [27].

## Results

### Study 1: A think-aloud study of completion of PAM-13 by adults with CF

**Fig. 2:**
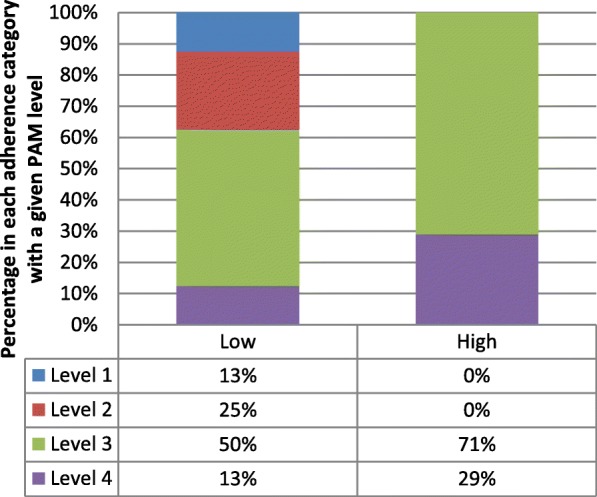
Percentage of PAM level by Low or High objective adherence in study 1

In study 1, the result showed that the majority of participants (12 out of 15) were at PAM Level 3 and above. Table 4 illustrates the PAM levels of activation of the participants. As can be seen, five of the eight participants who had low objective adherence were scored at Level 3 or Level 4 in the PAM-13. Figure 2 illustrate the percentage of participants in each adherence category with a given PAM level.

**Table 4:** PAM Levels of activation of the participants in study 1

PAM level	Participant ID	NO. of participants by objective adherence category	
		Low	High
Level 1	GL-LA-D02	1	0
Level 2	GL-LA-D01, PL-LA02	2	0
Level 3	GL-LA02, PL-LA01, PL-LA-D01, PL-LA-D02, GL-HA02, GL-HA-D01, PL-HA01, PL-HA02, PL-HA-D01,	4	5
Level 4	PL-HA-D02, GL-HA01, GL-LA01	1	2

The results of content analysis are presented in accordance with the a priori coding framework. Each code is elaborated with corresponding themes and examples of quotes. Table 5 illustrates the results of coding of the data relating to each item.

**Table 5:** Results of coding of the data relating to each item

	1	2	3	4	5	6	7	8	9	10	11	12	13
Code 1 Suitable item	✓	✓		✓		✓							
Code 2 Problematic content			✓					✓	✓	✓	✓	✓	✓
Code 3 Misinterpretation			✓		✓		✓		✓		✓	✓	

#### Code 1: Suitable item

No problems were identified for items 1, 2, 4 and 6. The data showed that participants had no difficulties in responding to these items, for example, “ *… and number two: taking an active role in my own health care is the most important thing that affects my health. Yes, again I agree and obviously my parents help a bit and sort of the hospital staff as well, so I will say I agree*” (GL-HA-D01).

Some participants indicated that completing the PAM-13 helped them to reflect on their health self-management, for example, *“It would help some of the patients; I dare say it has helped me a bit, doing it now, because it is some things you don’t think about. And you sit back and think about it while you are filling the form in.” (PL-HA-D02).*

#### Code 2: Problematic content

This code represents the data that participants questioned about the item content or wording or scale categories. There are five themes under this code, namely, lack of control over preventing problems, uncertainty resulting from co-morbidities, asking two things in one question, ambiguous wording and scale problem. The items involved in this code are Item 3, 8, 9, 10, 11, 12, and 13.

##### Theme 1: Lack of control over preventing problems

The data showed that participants found Items 3 and 11 were not suitable for people with CF. Participants reported that it is very difficult to prevent problems in CF as some conditions are unpredictable. Despite taking action to prevent problems, they may still find their conditions deteriorated. For example,

*“[Item 3: I am confident I can help prevent or reduce problems associated with my health] … I can’t prevent, but I can reduce sometimes … a difficult one, because with CF you can’t really have direct, you can’t have direct impact on what CF does to you. What it does it does, what you do with CF, what I do with my CF is kind of react to what it does... I want to say not applicable for me because I am not sure if I can reduce problems because CF is CF, I can do the physio which helps alleviate the symptoms a little bit, but the underlying condition, I don’t think I can change anything about it.” (PL-HA01)*

*“[Item 11: I know how to prevent problems with my health] … certain problems [I know how to prevent], and then there’s others I don’t know how to stop from happening. I think I’d have to put N/A for that one.” (PL-LA-D01)*

##### Theme 2: Uncertainty resulting from co-morbidities

Due to multiple co-morbidities of CF, participants indicated that their answers could be different in reference to different conditions and therefore they were unsure about which response to choose. The generalised content of items generates uncertainty for people with CF with multiple co-morbidities. For example,

*“[Item 8: I understand my health problems and what causes them.] This is only relating to CF, because I have got other health problems that impact on my CF, so I am not sure what the answer to that one, because I don’t really understand all my health issues and why they may affect my CF, because I have Meniere’s Disease as well and that strongly affects the medication I can take with CF, so that’s a hard one and the options I have got fall somewhere in between.” (GL-LA-D02)*

*“[Item 12: I am confident I can figure out solutions when new problems arise with my health.] I don’t know if that’s an agree or disagree to be honest. I think it depends what it is, what the problem is. For some problems yes I’m confident but others not so I don’t know that one … So if it’s something that you know about, if it’s a definite thing, for example, if it’s a normal CF chest problem then the solutions are you get some IV’s or some drugs or whatever it is that you know are available, that’s the solution or one of the solutions whereas I have nose problems as well, for example and as far as I know that’s it now, there’s nothing that can be done about it so I guess you can figure some solutions out but not everything’s got an answer to it.” (PL-HA02)*

##### Theme 3: Asking two things in one question

Participants suggested that Item 10 and 13 asked two different things in one question (i.e., eating right and exercising). They may agree with one thing but disagree with the other, which made it difficult to respond to the items. Participants with poor lung function indicated that it was impossible for them to do any exercise and therefore the exercising component of these two items was problematic for them. For example, participant PL-LA02 said that *“I am a bugger for exercising so I would agree only on the basis of one of those options, but actually I think there’s probably more than one question in there and that probably needs to be split out I think”.*

##### Theme 4: Ambiguous wording

Some participants found the wording of Item 9 and 12 vague and ambiguous. They indicated that they were unsure about what exactly the items were asking and therefore found it difficult to respond to. For example,

*“[Item 9: I know what treatments are available for my health problems.] I wasn’t quite sure what it really, you know so things like, as I said knowing about what treatments are available as well, is it asking about all possible treatments or is it just asking about the routinely available ones in your own hospital. I think it means the latter rather than everything that might be possible in the entire world.” (GL-LA01)*

*“[Item 12: I am confident that I can figure out solutions when new problems arise with my health.] What does that mean? On my own? That’s not a very well worded question. I don’t think, because I don’t know if that means I have to do it on my own or involve professionals, so I guess agree, but perhaps, well I am not clear.” (GL-HA01)*

Participants also pointed out that the wording (i.e., maintain lifestyle changes) in Item 10 and 13 seemed problematic. Participant PL-HA-D01 said *“That is a slightly confusing wording, because you can’t maintain a change. I suppose you can, but it is like, you can’t keep changing”.*

##### Theme 5: Scale problem

Some participants found the 4-point scale problematic (i.e., too few categories) and sometimes difficult to respond to.

*“I think it’s a bit ambiguous at best because you’ve got, you’ve basically got four options and I think most questions have a lot more than four options, so I think sometimes it’s hard to circle one of the four because in life things don’t neatly fall into options like that. So, I don’t find these that useful, in practice for myself, because what you tend to do in life is far more complexed than a set of options. That is like a set of one to four options. And my life doesn’t fall into categories.” (GL-LA-D02)*

#### Code 3: Misinterpretation

This code represents the data that participants either misunderstood the items or answered optimistically. The items involved in this code are Item 3, 5, 7, 9, 11 and 12.

##### Theme 6: Misunderstanding

This theme represents the situation when participants misunderstood the items and therefore answered a different question. For example,

*“[Item 9: I know what treatments are available for my health problems.] I am going to say disagree because things with the UK and US differ so much that sometimes you hear of a lot of treatments that just aren’t available here and vice versa.” (GL-LA02)*

This participant was considering availability of treatments in a very broad sense, including those that were not approved for use within the UK.

##### Theme 7: Answering optimistically

This theme represents the data that participants answered optimistically and their verbalised responses did not justify their answers. In particular, some participants pointed out that sometimes they *knew* what they should do but it does not necessarily mean that they would take action. As a result, they would agree with the items in theory but admitted that it may not be the case in practice. For example,

*“[Item 5: I am confident that I can tell whether I need to go to the doctor or whether I can take care of my health problem myself.] Agree strongly although just because I feel like I should go to the doctor, it doesn’t mean that I will, but I think especially when you’ve had something from birth you definitely get to know your own chest and symptoms. (Agree strongly)” (GL-LA02)*

*“[Item 3: I know how to prevent problems with my health.] Yes I would strongly agree, yes. I know how to prevent it, doesn’t mean I always do it, so yes, agree strongly (Agree strongly)” (PL-HA02)*

*“[Item 7: I am confident that I can follow through on medical treatments I may need to do at home.] I am going to put agree because whilst I can, the can do it and the actual doing it, it is, maybe the can is a bit ambitious because it’s, is it knowing what you have to do? I can do it, I know what I have to do with my nebulisers … or is it the, have the time to and that sort of thing … it could be seen as two different questions. Yes, I know what I am doing, I know what I should be doing, it is the fitting it in. so we’ve gone for agree.(Agree)” (PL-HA-D01)*

*“[Item 12: I am confident I can figure out solutions when new problems arise with my health.] Well I’d certainly like to think I could do that. But that’s alright in theory, it is just in practice really.(Agree)” (GL-HA-D01)*

*“[Item11: I know how to prevent problems with my health.] I suppose that’s similar to question 3. I probably interpret that in the same way as question 3 really, I agree. Again it is I know what I should be doing, whether that actually works is a different matter, or whether you can fit in what you should be doing.(Agree)” (PL-HA-D01)*

### Study 2: The association between PAM-13 and objectively measured adherence to nebuliser treatment

**Fig. 3:**
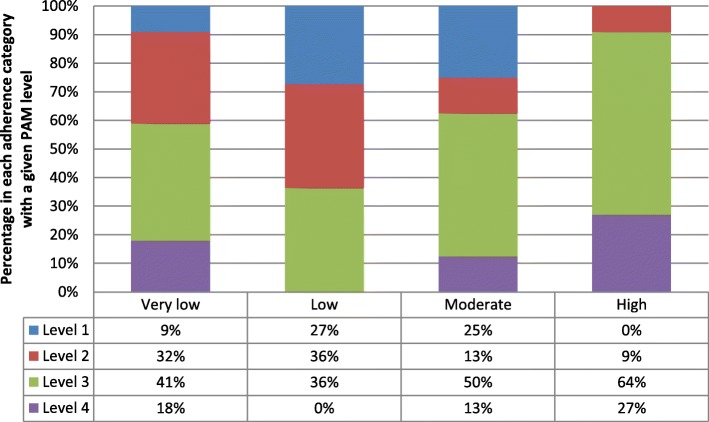
Percentage of PAM level by objective adherence category at baseline in study 2

**Fig. 4:**
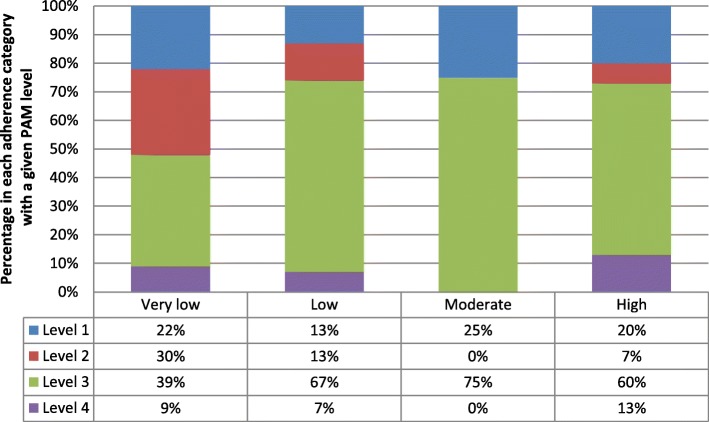
Percentage of PAM level by objective adherence category at follow-up in study 2

In study 2, the frequency of PAM level by objective adherence category at baseline and five-month follow up is illustrated in Table 6. To further demonstrate the PAM level composition of each objective adherence category, Figs. 3 and 4 show the percentage of participants in each objective adherence category with a given PAM level.

**Table 6:** Frequency of PAM level by objective adherence category at baseline and follow-up (5 months)

PAM level	Baseline objective adherence				Total	5 month follow up objective adherence				Total
	v.low	low	moderate	high		v.low	low	moderate	high	
	*n*	*n*	*n*	*n*	*n*	*n*	*n*	*n*	*n*	*n*
1	3	3	2	0	8	5	2	1	3	11
2	11	4	1	1	17	7	2	0	1	10
3	14	4	4	7	29	9	10	3	9	31
4	6	0	1	3	10	2	1	0	2	5
Total	34	11	8	11	64	23	15	4	15	57

Note: “v.low” means < 25%; “low” means 25.1–50%; “moderate” means 50.1–75%; “high” means > 75%

While we would expect to see low levels of PAM associated with low levels of objective adherence, this does not appear to be the case at either time-point. This was confirmed in an analysis using Cohen’s kappa which showed a lack of agreement between PAM levels and objective adherence category at baseline (*κ* = .03 (*95% CI*, −.09 to .15), *p* = .303) and at five-month follow-up (*κ* = .03 (*95% CI*, −.08 to .14), *p* = .292).

Using the continuous variables of PAM score and objective adherence percentage Pearson’s correlations also showed no relationship between PAM and adherence at baseline (*r* = 0.14, *p* = 0.28, *n* = 64) and follow-up (*r* = 0.06, *p* = 0.68, *n* = 57).

## Discussion

This research consists of two related studies investigating the patient activation measure (PAM) in patients with Cystic Fibrosis (CF), a long-term condition with important co-morbidities in which self-management is critical to optimum outcomes.

The findings of the ‘think aloud’ interviews (Study 1) have raised some concerns about the validity of using the UK PAM-13 in adults with CF to identify patients with high or low activation with the assumption that those who are highly activated are likely to be successful in managing their disease. These concerns may apply to other long-term conditions with multiple co-morbidities. Items within the PAM-13 asking about ‘preventing problems’ (Item 3 and 11) were identified as not being appropriate for patients with CF as some aspects of the condition and its progression are outside of the control of the individual. This also applies to patients with other long-term conditions, for example, Armstrong et al. [19] reported that patients with inoperable cancer and motor neurone disease found the item of ‘preventing problems’ inappropriate to their situation.

Items that asked about two things (i.e., eating right and exercising) in one question were also identified as problematic particularly because some patients with poor lung function have significant difficulties in exercising. This problem also pertains to other patients whose conditions limit physical activity exercise, and similar issues may arise for people with conditions which affect appetite and eating. According to Hibbard et al. [1], the PAM-13 was designed to be used in a wide range of chronic conditions. However, the present findings suggest that patients with certain conditions may find some items inappropriate or inapplicable to them.

The PAM-13 is designed to be applicable across conditions and therefore does not provide any context or guidance on which condition it is referring to and this means that patients with co-morbidities may find it difficult to respond to the items with certainty. Thus their answers may differ depending on to which condition they refer. This may cause confusion and misunderstanding when a healthcare professional is trying to infer a patient’s activation by reading the PAM-13 scores. A similar finding was reported by Armstong et al. [19] that patients with depression indicated they struggle to answer the PAM-13 as their self-care depends on their depression. Without clearer instruction or additional guidance from professionals, patients with multiple co-morbidities may not be able to provide informative and credible answers to the PAM-13.

Hibbard and Gilburt [11] indicate that “*patient activation captures not only the patient’s beliefs about their ability to self-manage but also the likelihood that they will put these beliefs into action*” (p.11), however our data suggests that this is not the case in this sample and indicates that one of the reasons for this is that participants sometimes responded according to what they thought they should do rather than what they actually did. This is consistent with a large body of data showing that there is a gap between intentions and behaviour such that around half (median 53%) of people who intend to perform a behaviour go on to perform the action [33]. Participants pointed out that a number of factors *(*i.e., social contexts, life situations, conditions, etc.) may constrain their action even though they ideally wanted to take the actions. While participants could be instructed to answer according to what they do rather than what they think they should do, this instruction is not currently included on the PAM-13 questionnaire and the official guidance for administering the PAM-13 emphasises standardised administration.

The ‘think-aloud’ study showed that patients with CF encountered a number of problems and difficulties when answering the PAM-13, which raised the question of whether patients need additional help to fill in the PAM-13 in order to obtain an informative and credible outcome. Chew et al. [17] conducted an ethnographic study of the implementation of the PAM and they suggested that standardised administration of the PAM was challenging and may exclude certain patient groups. Our findings support Chew et al.’s [17] argument. If the PAM-13 is to be used as a research tool *(*e.g., evaluate interventions) or in situations where careful direction or additional help is not possible, the results of the PAM-13 need to be interpreted with caution.

The wording of the UK version PAM-13 is different from the original PAM-13. When Ellins and Coulter [12] first introduced the PAM-13 to the UK, they anglicised some wording of items, and this anglicisation included changes to some of the items that participants found most challenging to answer. The wording was modified to make them more suitable for telephone survey, although it is not clear whether these items are appropriate for paper-and-pen test and whether this UK version has been fully validated. Based on our ‘think-aloud’ data, it may be that the original US wording of Items 3, 10 and 13 may be more appropriate than the modified UK version. More empirical evidence is needed to verify the modified wording of the UK version PAM-13.

The quantitative results (Study 2) are consistent with the findings of the ‘think aloud’ interviews (Study 1). The results show no significant correlation between the PAM-13 scores and objective measures of nebuliser adherence in adults with CF at either the baseline or the follow-up point. Most of the previous studies that have demonstrated evidence of the positive relationship between PAM levels and medication adherence have been based on self-reported adherence measures, which are known to be subject to self-report bias [19]. Evidence of an association between PAM scores and objectively measured self-care behavioural data is less convincing. Shah et al. [32] used PAM and clinical parameters (i.e., mean levels of A1C, fasting blood glucose, BMI, total cholesterol, and triglycerides) to evaluate an intervention programme. They reported that changes in clinical parameters were not correlated with either PAM level at baseline nor change in PAM level from baseline to 6 months [32]. Likewise, Mayberry et al. [34] found Patient Activation was unrelated to glycemic control among adults with Type 2 diabetes. These findings concur with the present study, suggesting that PAM scores are not necessarily related to objectively measured data of self-care.

### Limitations

Both studies in this research were relatively small-scale and focused on a specific patient sample, i.e., adults with CF. Given the specific characteristics of CF, generalisation of the findings to other chronic conditions should be with caution. Nonetheless, the findings show that some items might be problematic for patients with some long-term conditions or co-morbidities. Future studies may seek to evaluate the PAM-13 in other patient populations with long-term conditions and multiple co-morbidities.

### Implications

The PAM-13 is currently being used in a number of settings, for example, as a tailoring tool to inform health coaching and service delivery; and as an outcome measure to evaluate care provision [19]. While PAM serves as a useful tool to predict outcomes associated with self-care in cross-sectional studies with large sample size, its application in individual cases should be carried out with caution. The findings in our study suggest that in some situations the activation data based on PAM may be unreliable and may result in support being inappropriately targeted. It is therefore vital that health professionals are aware of these potential discrepancies and where possible utilise objective measures of self-care such as objective adherence data to verify the PAM level categorisation. Health professionals should also be aware of the difficulties that some items might pose and provide patients with support and guidance to answer appropriately.

## Conclusions

This research investigated the role of patient activation in medication adherence of patients with CF. Instead of relying on self-report medication adherence as most of previous studies did, this research adopted objectively measured medication adherence which is more accurate than self-report data. The results suggested that patient activation as measured by the UK PAM-13 was not reliably associated with objectively measured adherence to medication. This may well result from difficulties in responding to specific items and because some participants responding according to what they knew they should do, rather than what they actually did. It seems likely that patients with similar long-term conditions and patients with multiple co-morbidities, may also find it difficult to respond to the PAM-13. Further research is therefore needed to verify the validity and reliability of the UK version PAM-13 in different patient populations.

## Data Availability

The datasets analysed during the current study are available from the corresponding author on reasonable request.

## Abbreviations

A1C: Glycated hemoglobin
ACtiF: Adherence to treatment in adults with Cystic Fibrosis
BMI: Body Mass Index
CCGS: Clinical Commissioning Groups
CF: Cystic Fibrosis
CFHealthHub: A web platform that displays nebuliser adherence data, developed and tested as part of NIHR Grant Reference Number RP-PG-1212-20015: ‘Development and evaluation of an intervention to support Adherence to treatment in adults with Cystic Fibrosis (ACtiF)
FEV1: Forced expiratory volume in the first second
NHS: The National Health Service
NIHR: National Institute for Health Research
PAM-13: Patient activation measure
Qualcomm: Quality communications
UK: United Kingdom
US: United States

## Footnotes

1 Nebuliser adherence data were used to indicate high or low adherence. Nebuliser adherence data were objectively captured with chipped devices (E-track®). Adherence was calculated as a percentage between total amount of medication used against the agreed dose between clinicians and adults with CF and clustered according to a pragmatic algorithm-based clustering method [27]. The most recent 12-month of data in the period from 01 Jan 2016 to 31 Mar 2017 for each potential participant was used for clustering (very low adherence < 25%; low adherence 25.1–50%; moderate adherence 50.1–75%; high adherence > 75%) [27].

2 Participants’ lung function was defined by their FEV1. The best % predicted FEV1 (calculated with Knudson equation) [28] from 01 Jan 2016 to 31 Dec 2016 for each potential participant was used. % FEV1 ≥ 70% was classified as high (i.e., good lung function), which is the standard cut-off used in CF epidemiological studies [29]. Similar cut-off also applies well for the UK data [30]. Conversely, % FEV1 < 70% was classified as low (i.e., poor lung function).

3 The first two-week period was at baseline (i.e., 14 days post-consent); while the second two-week period was at the five-month follow up (i.e., 14 days preceding the completion of follow-up questionnaires).
